# Feasibility and acceptability of a culturally tailored physical activity intervention for Arab-Australian women

**DOI:** 10.1186/s12905-021-01250-3

**Published:** 2021-03-30

**Authors:** Aymen El Masri, Gregory S. Kolt, Emma S. George

**Affiliations:** 1 School of Health Sciences, Western Sydney University, Locked Bag 1797, Penrith, NSW 2751 Australia; 2 Translational Health Research Institute, Western Sydney University, Penrith, Australia

**Keywords:** Arab women, Migrant, Physical activity, Feasibility study

## Abstract

**Background:**

Despite being one of the largest migrant groups in Australia, few physical activity interventions exist for Arab-Australians. The primary aim of this study was to test the feasibility and acceptability of a 12-week culturally tailored physical activity intervention for Arab-Australian women.

**Methods:**

This study used a single-group pretest–posttest design, and was informed by extensive formative research and consultation involving the Arab-Australian community. Participants were insufficiently active Arab-Australian women aged 35–64 with no current illness or injury that would limit physical activity participation. The intervention comprised 6 face-to-face physical activity and education sessions over 12 weeks. The intervention was conducted at 2 separate intervention sites in Western Sydney, Australia. Feasibility outcomes included recruitment, session attendance, and retention. The acceptability of the intervention was assessed through a process evaluation questionnaire completed post-intervention. Accelerometers and the short-form International Physical Activity Questionnaire were used to measure physical activity at baseline and post-intervention. Descriptive statistics were used for feasibility and acceptability outcomes, and changes in physical activity were examined using Wilcoxon signed-rank tests.

**Results:**

Of the 53 women who were contacted or expressed interest, 22 were eligible and enrolled in the study. Participants were primarily recruited through direct contact by Arab-Australian community workers and by word-of-mouth. Average session attendance was 63% and the retention rate post-intervention was 68%. The culturally-related intervention components, such as the appropriateness of content, and women-only setting, were rated highly favourably (4.33 to 4.87/5). General intervention elements, such as the face-to-face delivery, knowledge and approachability of facilitators, and session structure, were also rated favourably (4.33 to 4.93/5), and the lowest scored item was the intervention session frequency (3.2/5). There were no statistically significant changes in physical activity post-intervention.

**Conclusions:**

The findings from this study highlighted factors related to recruitment and delivery that need to be considered when developing physical activity interventions for Arab-Australian women. Further research is required using a larger sample and a randomised controlled trial design to examine the longer-term impact on physical activity, and to also examine ways of increasing intervention engagement and retention among Arab-Australian women.

*Trial Registration*: ANZCTR, ACTRN12618001392257. Registered 20 August 2018, https://www.anzctr.org.au/Trial/Registration/TrialReview.aspx?id=375636.

## Background

It is known that regular physical activity reduces the risk of all-cause mortality and various chronic diseases, such as cardiovascular disease and diabetes [1]. Despite this, it has been reported that more than a quarter of adults aged 18 years and older are not meeting physical activity guidelines [2]. Evidence suggests that migrant populations tend to engage in lower levels of physical activity in comparison to non-migrant populations [3, 4].

There are many factors that influence the physical activity levels of culturally and linguistically diverse (CALD) or migrant populations such as the migratory experience, limited knowledge on preventative health benefits of physical activity, language, culture and religion, and socioeconomic status [5, 6]. For example, it has been reported that physical activity levels are often higher among CALD groups when in their country of origin, due to a higher level of incidental physical activity as opposed to other modes of transport such as cars which is the norm in host countries [7]. Cultural and religious practices can also pose as a potential barrier to the physical activity levels of CALD populations [5, 8, 9]. However, the factors influencing physical activity differ between CALD groups [10].

As a result of civil unrest in the Arab world [11], there are large numbers of Arab migrants residing outside their country of origin. Arab migrants are those with ties to Arabic speaking countries in the Middle East (e.g., Lebanon, Syria) and North Africa (e.g., Egypt, Morocco). Evidence suggests that Arab migrant populations in the diaspora appear to display higher rates of certain chronic diseases when compared to those of the host population. For example, rates of diabetes are higher among Arab migrant populations in Australia [12], Denmark [13], Sweden [14], and the United States (US) [15] in comparison to the majority population of the host country. Additionally, a higher body mass index (BMI) among Lebanese migrants in Australia [16] and higher levels of obesity among Iraqi migrants in Sweden [14] in comparison to those born in their respective host country have also been reported. Despite this, lower levels of physical activity among Arab migrants when compared with the majority population of the host country in various contexts have been reported, for example among Lebanese populations in Australia [17], Iraqi migrants in Sweden [14, 18], and Arab-Americans in the US [19]. Moreover, low levels of physical activity and a higher BMI have also been reported to be an issue among Arab populations in their country of origin [20].

A potential explanation for the low levels of physical activity among Arab migrant populations could be attributed to their specific experiences, needs, and requirements concerning physical activity participation. Commonly reported factors influencing the physical activity levels of Arab migrant populations [7, 21–23] and Arab populations in their country of origin [24] that are shared with the general population of the host country include a lack of time, existing health conditions, and social support. Other factors that may be more specific to Arab migrant populations can include traditional gender roles, whereby women take on the majority of domestic chores leaving little time for physical activity [7, 21, 22]. Further, there is a requirement for culturally appropriate activities and gender-exclusive settings for physical activity [7, 21–23, 25]. Due to the unique needs and preferences of Arab migrant populations, health promotion initiatives designed for this population need to be culturally tailored.

Australia is a culturally diverse country, with 26% of the Australian population having been born overseas [26]. Arab-Australians are one of the larger CALD groups in Australia. In 2016, there were 259,114 Arab-Australians residing in New South Wales (NSW), the most populous state in Australia, which was a 20.2% increase in comparison to 2011 [27].

To our knowledge, there are few physical activity initiatives targeting Arab migrant adults, particularly in an Australian context. Many of these initiatives have generally adapted existing programs for implementation with Arab migrants [28–30], physical activity was not a primary outcome [29–31], and only one study was conducted in Australia [31]. These interventions were all culturally tailored to meet the needs and preferences of the target population, which included participatory methods to inform and tailor the intervention [29, 31], culturally and gender-matched intervention personnel [28–30], gender-exclusive settings for physical activity [29, 30], and culturally tailored content (e.g., Arabic sayings and themes) [28–31]. The level of detail on reporting such adaptations varied. It is important there is transparency with reporting the cultural adaptations made in interventions when targeting CALD populations as it allows researchers to replicate intervention aspects that were found to be successful for future health promotion initiatives [32].

There is a strong need for the development of robust physical activity interventions designed specifically for Arab-Australians. One critical step in developing and optimising such interventions is to test the feasibility of the intervention as an initial step before larger-scale replication [33]. The aim of this study was to develop and test the feasibility and acceptability of a culturally tailored physical activity intervention for Arab-Australian women aged 35–64 years. A secondary aim, which was exploratory in nature, was to test the preliminary efficacy of the intervention on physical activity levels, self-efficacy for physical activity, and anthropometric outcomes.

## Methods

This study was approved by the Western Sydney University Human Research Ethics Committee (H12725) and the trial was prospectively registered with the Australian New Zealand Clinical Trials Registry (ACTRN12618001392257).

### Trial design

This study was guided by the CONSORT statement for reporting pilot and feasibility trials [34]. This study used a single-group pretest–posttest design. The intervention was conducted at two separate community centres in Western Sydney, Australia. The main objective of this study was to assess the feasibility, which included effectiveness of recruitment strategies, attendance, and retention, and to assess intervention acceptability through a process evaluation. A secondary aim, which was exploratory in nature, was to test the preliminary efficacy of the intervention on physical activity levels, self-efficacy for physical activity, and anthropometric outcomes. As the primary aim of this study was focused on testing the feasibility and acceptability, no sample size calculation was performed.

### Participants

Participants were eligible for inclusion in this study if they met the following criteria: (1) female, (2) identify as Arab-Australian, (3) aged 35–64 years, (4) not currently sufficiently active (i.e., not currently engaging in 150 min of moderate-vigorous intensity physical activity over 5 or more days each week), and (5) no current disease or illness that would limit participation or put them at further risk of aggravating their condition with physical activity participation. Participant eligibility was assessed by intervention personnel and community workers verbally. No language requirement was set for inclusion in the study.

### Formative work

The design and development of the intervention was informed by extensive preliminary research, which included a systematic review of physical activity interventions among CALD populations [32], a systematic review of qualitative studies exploring the factors influencing physical activity participation among Arab migrants [35], an epidemiological study examining the odds of lifestyle-related chronic diseases among Lebanese-Australians and the influence of physical activity and sitting time on these outcomes [12], and a qualitative study exploring the experiences, barriers, and enablers to physical activity and sedentary behaviour among Arab-Australians aged 35–64 years [22]. To develop the intervention, ongoing community consultation and informal discussions were organised with members of the Arab community. A meeting was conducted to review the proposed intervention which included the PhD candidate (AE), supervisory panel member (ESG), and members of the Arab-Australian community with experience working with the target population: a qualified physiotherapist (female Arab-Australian, cofacilitated the intervention), and a social worker from one centre where the intervention took place (female Arab-Australian). All intervention components were discussed to ensure they were scientifically and culturally appropriate, and only minor modifications were made to the intervention protocol.

### Recruitment and data collection

Recruitment occurred in two phases. Initial recruitment was through the distribution of flyers (e.g., within community centres), social media (Facebook) posts through community organisations, verbal presentations at community centres in Western Sydney, and word-of-mouth. The second phase was through direct contact (i.e., face-to-face, telephone) by a social worker at site one and an accredited dietitian at site two. Only community members who were known to be of Arab ethnicity and aged between 35 and 64 years were contacted. One intervention site specifically catered to Arab populations and the other was a Women’s Health Centre catering to the local public. The social worker and dietitian were both Arab-Australian (Lebanese ethnicity) and were all known to the participants through various other services offered at their respective sites. Direct contact methods are an important recruitment strategy and also beneficial for establishing trust [36, 37]. Trust is especially important when working with Arab migrants, as some are reluctant to engage with ‘outsiders’ or people not known to them [36, 37].

Those who were interested in participating were screened for eligibility. Eligible participants were then invited to attend an information and baseline measurement session at the respective intervention site where they were given a brief on the intervention and provided with a participant information sheet and consent form. Participants were asked to complete the compulsory section of the Exercise and Sport Science Australia [38] adult pre-exercise screening system to identify those who may be at risk of harm or injury from physical activity participation. Although the physical activity sessions in this study were of light-moderate intensity, any participant who indicated a previous condition on the pre-exercise screening tool were asked to provide medical clearance from a medical practitioner prior to participation in the intervention. Prior to providing written consent participants were provided with the opportunity to ask any questions about the program or to raise any concerns they might have had.

Participants were asked to complete a series of questionnaires (available in English and Arabic) which included questions on demographic characteristics (i.e., age, education, income, ethnicity, country of birth, religion, and year of arrival in Australia if applicable), the short-form IPAQ [39], and a modified Self-Efficacy for Exercise (SEE) scale [40]. They were also fitted with an accelerometer to wear for a 7-day period. The short-form IPAQ includes questions to assess physical activity (i.e., walking, moderate, and vigorous physical activity) and sitting time. The SEE was originally designed to assess self-efficacy for exercise, however, the survey was adapted through text changes in this study to assess self-efficacy for physical activity, which has been similarly done in other studies [41–43].

Trained female research assistants recorded weight measurements using a calibrated digital scale, height using a portable stadiometer, and waist circumference measurements using a non-elastic tape measure, level with the umbilicus [44]. Each measurement was recorded at least twice, with a third measurement only recorded if there was a discrepancy greater than 0.5 kg for the two weight measurements and 0.5 cm for the two height and waist circumference measurements. The average of these measurements was calculated and used in the analysis. At the conclusion of the 12-week intervention period, all participants were invited to attend a post-intervention data collection session. At these sessions, participants were again provided with an accelerometer to wear for 7 days, had their weight and waist measurements recorded, and were asked to complete a series of questionnaires including the short-form IPAQ, SEE, and a process evaluation questionnaire.

The intervention was conducted with separate groups at two community sites in South Western Sydney, Australia, both of which are not-for-profit and offer a range of free services to the community. South Western Sydney is known to be a culturally diverse region, with a higher proportion of people born overseas (43%) in comparison to that of the NSW population (34%) [45]. The intervention was delivered from April to July 2019 for the first group and between August-November 2019 for the second group. Ramadan coincided with the scheduling for the first group, with two face-to-face sessions being scheduled during this period. Observers of Ramadan abstain from food and drink from sunrise until sunset (approximately 11–12 h in May–June 2019 in the geographic region of this study). Although observers of Ramadan may feel less energised than normal while fasting, research suggests that physiological responses of carrying out physical activity during Ramadan were within normal ranges, thus physical activity during Ramadan can be performed without posing health risks [46]. There is also evidence to suggest that being physically active during Ramadan can be beneficial for health [47], and this message was emphasised in the weeks leading up to Ramadan. To account for participants’ lower energy levels during sessions that coincided with fasting, lower intensity physical activity (e.g., Pilates) was requested and scheduled during this period. One scheduled session during the final week of Ramadan (the week before Eid, a festival marking the end of Ramadan) was rescheduled due to limited participant availability.

### Intervention

The intervention consisted of six face-to-face sessions (once every 2 weeks) over a 12-week period. The sessions were scheduled during daylight hours and included an educational and a practical physical activity component, and lasted for approximately 1.5–2 h. The social cognitive theory (SCT) was used to inform intervention components in order to facilitate behaviour change, as the SCT is one of the most widely used and empirically-supported theories to explain and predict physical activity [48]. The educational component was cofacilitated by the lead investigator who is a male Arab-Australian (to ensure intervention fidelity) and a female Arab-Australian physiotherapist. For the physical activity component, participants engaged in a range of different activities, which included light-moderate intensity physical activity (e.g., dancing/Zumba), flexibility and balance type physical activity (e.g., Pilates), and light-moderate strength-based physical activity. The physical activity sessions were facilitated by the female Arab-Australian physiotherapist in female-only settings, with guest instructors to assist with some sessions (e.g., Zumba).

The intervention was scheduled once every 2 weeks in an attempt to promote accountability and to allow time to apply the strategies learnt in the previous sessions. Although the greatest health benefits are obtained from performing 150 min of moderate-vigorous physical activity across five or more days per week, there is evidence to suggest that even lower levels of physical activity are beneficial for health in comparison to no physical activity [49]. Therefore, a key message incorporated in this intervention was that any physical activity is better than no physical activity, and participants were encouraged to perform physical activity as much as possible beyond the structured intervention. All participants were provided with a program pack, which included a university branded hat, drink bottle, and a towel.

When designing and delivering a physical activity intervention for CALD populations, it is important that the program or intervention offered is culturally tailored to suit the needs and preferences of the target population [32]. Female data collection personnel were used at baseline and post-intervention, with the majority being bilingual Arab-Australian women. Female-only settings for the physical activity component were provided due to privacy requirements of some Arab women [22]. The program material was available in Arabic and English, however the presentations were delivered in Arabic as this was the primary language spoken by all participants. The intervention was conducted at venues familiar to participants and community workers known to participants were in attendance at all intervention sessions. The program was given a culturally-relevant title ‘Al-Harakeh Barakeh’ (‘Movement is a blessing’), which is a commonly used Arabic saying to signify the health benefits of physical activity. Further, the intervention included culturally-relevant intervention material (e.g., images, all sessions incorporated aspects of Arab culture directly relating to the topic and findings relevant to Arab migrant populations, epidemiological data on Arab migrant populations concerning physical activity and health, Arabic idioms, sayings, and themes). Barriers and enablers to physical activity identified in an earlier study conducted among Arab-Australians guided session topics and discussions within each session [22]. As social support has been identified as an important enabler to physical activity for Arab-Australian women [22], the intervention was group-based. As recommended by community workers at one centre involved in the intervention and in line with previous research reporting that Arab women often have familial responsibilities, such as looking after children [7, 22], the intervention was scheduled during school hours which enabled many women to attend. The intervention schedule is presented in Table 1.

**Table 1:** Intervention schedule, topics, and activities

Session	Topics and activities
Session 1 Introductory session	Introduction to program
	What is physical activity and sedentary behaviour? Participants receive an individualised physical activity report based on accelerometer measured physical activity
	The health of Arab-Australians (e.g., general health, physical activity levels, cultural factors influencing physical activity participation)
	SMART goals (discussion and setting short and long-term goals)
	Physical activity session—warm up, light-moderate intensity strength-based activities, cool down
Session 2 Physical inactivity versus sedentary behaviour and the influence of traditional gender norms on physical activity	The distinction between physical inactivity and sedentary behaviour
	Traditional gender roles and its impact on physical activity participation
	Discussion on strategies to increase physical activity—discussions based on current and previous barriers encountered
	Revisiting SMART goals
	Physical activity session—warm up, Pilates, cool down
Session 3 Interpersonal support and maintenance for physical activity	Interpersonal support and physical activity
	Maintenance—discussion on strategies for maintenance and discussion on any self-reported changes in physical activity and sedentary behaviour habits (progress monitoring)
	Strategies to increase physical activity—discussions based on current and previous barriers encountered
	Physical activity session—warm up, Dancing/Zumba, cool down
Session 4 Chronic disease and physical activity among Arab-Australians	Chronic disease among Arab-Australians—discussion of rates of chronic disease experienced among Arab-Australians (comparisons made by physical activity level and with non-Arab populations)
	Physical activity among Arab-Australian populations—discussion on impact of acculturation, potential reasons for physical activity decline, aspects of Arab culture influencing participation, and ways to address physical activity decline
	Strategies to increase physical activity—discussions based on current and previous barriers encountered
	Physical activity session—warm up, light-moderate intensity strength-based activities, cool down
Session 5 Mental health and motivation	Physical activity and mental health—discussion of the benefits of physical activity towards mental health and reasons why physical activity makes us feel better. Discussion on physical activity and stress/stress management
	Motivation for physical activity—discussion on time management, overcoming laziness, and sustaining motivation in the long-term
	Strategies to increase physical activity—discussion based on current and previous barriers encountered
	Physical activity session—warm up, Pilates, cool down
Session 6 Conclusion of intervention and long-term maintenance	Program revision—revision quiz and recap of topics covered throughout program
	Revisiting SMART goals and preparation for post-intervention physical activity maintenance
	Group discussions and interactive activities—recap strategies learnt and implemented and also discussions on long-term physical activity maintenance
	Q and A—opportunity for participants to ask questions about any topics covered
	Physical activity session—warm up, Dancing/Zumba, cool down

### Feasibility outcomes and process evaluation

To assess the feasibility and acceptability of the intervention, a range of outcomes were measured. These included recruitment source (e.g., direct contact, word-of-mouth), attendance at each of the six sessions, participant retention from baseline to post-intervention data collection, the availability of complete data for all outcomes, and a subjective process evaluation of intervention components to assess acceptability.

Participants were asked to complete an evaluation of the intervention through a process evaluation questionnaire (Arabic or English) completed at the conclusion of the 12-week intervention. This survey sought participants’ perspectives on each aspect of the intervention, including the cultural relevance of intervention components, the acceptability of general intervention components, perceived benefits associated with the program, and their overall satisfaction with the program. The survey included 12 questions, which included five multiple choice questions with opportunity for further comment, a series of statements with Likert scale responses (scoring ranging from strongly disagree = 1 to strongly agree = 5) addressing four intervention components, and three open-ended questions asking participants what they enjoyed the most and least, including a question requesting information as to how the program could be improved.

### Data analysis

Descriptive statistics were used to explore the demographic characteristics of the sample, and for the feasibility and acceptability outcomes. Due to the small sample, a conservative approach was adopted by using the nonparametric Wilcoxon signed-rank test as it cannot be guaranteed that the difference in outcomes from baseline to post-intervention follow a normal distribution. Wilcoxon signed-rank tests are an alternative to *t* tests for data that is not normally distributed and were used to examine changes in the outcomes, which were accelerometer-measured physical activity (daily average % wear time, minutes in moderate-vigorous physical activity accumulated in 10-min bouts, step count), self-reported physical activity (MET minutes per week), self-efficacy for physical activity (0–90), weight (kg), waist circumference (cm), and body mass index (BMI; kg/m^2^) from baseline to post-intervention [50]. Missing observations were excluded from the analysis, except where detailed protocols regarding the handling of missing or invalid data were available for instruments that were used in this study (e.g., the short-form IPAQ). For the short-form IPAQ, observations were excluded from the analysis if a participant had missing or invalid data for one type of physical activity. Accelerometer wear-time validation was conducted using the Choi et al. [51] algorithm and scored using Troiano et al. [52] cut-points. Valid accelerometer data for this study was set at ≥ 10 h per day across ≥ 4 days, as suggested in earlier research [53]. Data analysis was performed by AE using SPSS 24 and the *p* value was set at 0.05.

## Results

**Fig. 1:**
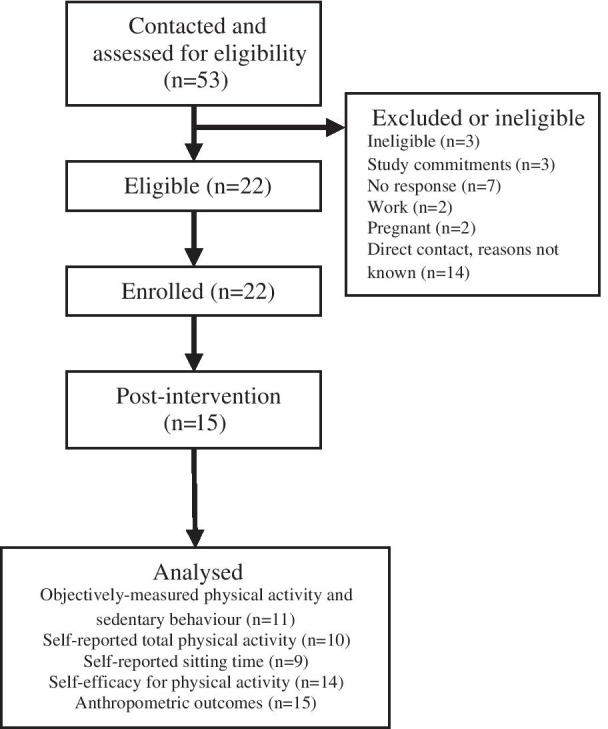
Participant flow

A total of 53 Arab-Australian women were contacted or expressed interest in participating in the study. Of these, 22 participants were deemed eligible and enrolled in the study, of which 15 were retained post-intervention. A total of 8/13 (62%) and 7/9 (78%) were retained at the first and second site, respectively (see Fig. 1 for participant flow).

Table 2 includes demographic details of the participants at baseline. Participants were 22 Arab-Australian women with a mean (SD) age of 48.23 (± 9.33) years. For baseline anthropometric values, mean BMI was 32.26 (± 5.56) kg/m^2^, mean weight was 81.40 (± 13.45) kg, and mean waist circumference was 97.06 (± 11.90) cm. Almost half of the participants were of Lebanese ethnicity (45.45%), followed by those of Syrian ethnicity (22.73%). A total of 20 participants were born overseas and had lived in Australia for 19.80 (± 12.98) years. Most participants were of the Muslim faith (86.36%). A large proportion of the sample reported having a university degree (40.90%), followed by a certificate/diploma (22.73%).

**Table 2:** Demographic characteristics

Variable	n (%)
Education	
No school certificate	3 (13.64%)
School certificate	2 (9.09%)
High School certificate	3 (13.64%)
Certificate/diploma	5 (22.73%)
University degree or higher	9 (40.91%)
Yearly household income	
< $9999	4 (18.18%)
$10,000–$19,999	5 (22.73%)
$20,000–$29,999	1 (4.55%)
$30,000–$49,999	2 (9.09%)
$50,000 or more	1 (4.55%)
Prefer not to answer	8 (36.36%)
Religion	
Muslim	19 (86.36%)
Christian	3 (13.64%)
Languages spoken at home	
Arabic	13 (59.09%)
English	1 (4.55%)
Arabic and English	8 (36.36%)
Ancestry	
Lebanese	7 (31.82%)
Syrian	5 (22.73%)
Palestinian	1 (4.55%)
Jordanian	1 (4.55%)
Egyptian	3 (13.64%)
Libyan	1 (4.55%)
Sudanese	1 (4.55%)
Algerian	1 (4.55%)
Lebanese and Australian	2 (9.09%)
Country of birth	
Australia	2 (9.09%)
Lebanon	6 (27.27%)
Syria	5 (22.73%)
Palestine	1 (4.55%)
Jordan	1 (4.55%)
Egypt	3 (13.64%)
Kuwait	1 (4.55%)
Libya	1 (4.55%)
Sudan	1 (4.55%)
Alegria	1 (4.55%)

## Intervention feasibility

### Recruitment and retention

A range of recruitment methods were employed for this study, however, the most effective approach was through direct contact from community workers at each respective intervention site (91%). Data from the process evaluation indicated that most participants reported finding out about the program through a community centre (53%), followed by referral from a friend (40%), and then social media (13%).

A total of 15 participants (68%) were retained post-intervention. Three participants did not return after the first session. Of those who dropped out, one was due to personal matters and one due to work commitments. Other participants did not respond to contact (e.g., phone call, text messages from community centre personnel). The average session attendance at the first site was 59%, with a range of 38–100%. Session attendance at the second site was 71%, with a range of 44–100%.

### Accelerometer compliance

A total of 14/22 (64%) participants at baseline had valid accelerometer data. Among those who were retained following the conclusion of the intervention, a total of 11/15 (73%) participants had valid accelerometer data. At post-intervention, two participants did not have any data recorded on their accelerometer, one participant had data only recorded across 2 days, and a further participant returned a malfunctioning accelerometer on which no data was recorded. The participant who returned the malfunctioning accelerometer reported that she only wore the accelerometer for 2 days, which would have meant that any recorded data would have been considered invalid. A total of 11 participants had complete data that were used in the analysis.

### Intervention acceptability

The main reasons reported by participants for joining the program included ‘health’ (67%) or ‘to get motivated to improve my lifestyle’ (67%), ‘to lose weight’ (47%) and ‘increase physical activity’ (47%), and ‘my friend recommended it to me’ (13%).

The main factors that prevented or made it difficult for participants to attend sessions were Ramadan (60%), caring for children (20%), familial responsibilities (20%), medical appointment or sickness (20%), lack of motivation (13%), time (13%), shyness (13%), work (13%), study (13%), and a lack of social support (7%). Factors that encouraged participants to attend sessions were to improve health (100%), women-only setting (73%), free of costs (67%), location/setting (60%), and social support (47%). The results of the process evaluation are presented in Table 3, which presents the average rating of intervention components by study completers.

**Table 3:** Results of the process evaluation questionnaire

	Mean	SD	Range
Culturally-related intervention components			
The content and material were relevant to Arab Australian women	4.80	0.41	4–5
The program facilitators were respectful of my culture and beliefs	4.80	0.41	4–5
Having the program delivered in Arabic made me more engaged in the program	4.40	0.83	3–5
Co-facilitation of the education sessions by a male facilitator did not bother me	4.33	1.11	1–5
The activities in the practical physical activity sessions were appropriate for Arab-Australian women	4.87	0.35	4–5
Having the program at a familiar venue facilitated my engagement with the program	4.87	0.35	4–5
Program facilitators were culturally aware of the Arab-Australian culture	4.67	0.62	3–5
Having the facilitators of Arab-Australian background was very beneficial for my engagement in the program	4.53	0.74	3–5
I prefer a women-only program	4.67	0.49	4–5
General intervention components			
Group sessions were preferred over individual or online sessions	4.67	0.49	4–5
Program facilitators were knowledgeable	4.67	0.49	4–5
Program facilitators were supportive and approachable	4.80	0.41	4–5
The physical activity sessions were enjoyable	4.93	0.26	4–5
The physical activity sessions catered to women of all abilities	4.80	0.41	4–5
I liked the structure of each session (education followed by physical activity)	4.80	0.41	4–5
The scheduling of the sessions did not impact on my other commitments (e.g., caring for children)	4.60	0.63	3–5
One session per fortnight was sufficient	3.20	1.37	1–5
The session duration was appropriate (90mins per session)	4.33	0.98	2–5
The venue was easily accessible	4.67	0.49	4–5
The venue was appropriate	4.73	0.46	4–5
I felt like I was a member of the group	4.73	0.46	4–5
I had a good relationship with other women in the program	4.80	0.41	4–5
Perceived benefits of program			
I have obtained valuable knowledge I was not previously aware of	4.53	0.52	4–5
I have learnt new skills related to physical activity	4.73	0.46	4–5
I feel better in myself	4.60	0.51	4–5
I am happier	4.67	0.49	4–5
I have more confidence	4.47	0.64	3–5
I am more active with my family	3.93	1.22	2–5
I now set physical activity goals	4.43	0.76	3–5
I now track my physical activity	4.40	0.74	3–5
Satisfaction with program			
I am satisfied with the program	4.87	0.35	4–5
My involvement in the program was enjoyable	4.80	0.41	4–5
I would recommend this program to others	4.87	0.35	4–5

*SD* standard deviation

For questions concerning the culturally-related intervention components, the mean rating was 4.66. The two equally highest scored responses were ‘The activities in the practical physical activity sessions were appropriate for Arab-Australian women’ and ‘Having the program at a familiar venue facilitated my engagement with the program’ (4.87). The lowest scored response, however, still rated highly favourable, was to the statement ‘Co-facilitation of the education sessions by a male facilitator did not bother me’ (4.33). Participants reported in the open-ended questions that the delivery of the program in Arabic was positive, and they felt ‘comfortable’ or that the facilitator made them feel comfortable. However, one participant noted that ‘a female facilitator would be great’.

The average rating for the general intervention components was 4.59. The highest scored response was to the statement ‘The physical activity sessions were enjoyable’ (4.93). The lowest scored response regarding the general intervention components was to the statement ‘One session per fortnight was sufficient’ (3.20). Responses to open-ended questions regarding the general intervention components indicated that participants wanted more frequent sessions, preferably weekly.

The average rating for the perceived benefits of the program was 4.47. For the perceived benefits of the program, the statement ‘I have learnt new skills related to physical activity’ had the highest average score (4.73) and the statement ‘I am more active with my family’ had the lowest average score (3.93).

Scores related to the participants’ satisfaction with the program (4.87), enjoyment with the program (4.80), and if they would recommend the program to others (4.87) were highly favourable. All participants (100%) indicated that their physical activity levels had ‘greatly increased’ or ‘increased’ and the majority (93%) indicated that their sitting time had ‘greatly decreased’ or ‘decreased’.

In the open-ended questions regarding aspects of the program that participants liked or most enjoyed, the most commonly cited response was the physical activity sessions, the social aspect of the program (e.g., making new friends), and the usefulness and simplicity of the information. Other aspects of the program that the participants reportedly enjoyed or liked about the program included the Arabic speaking facilitators, that the intervention was conducted at an Arabic community centre, the program was free, and the interactive presentations. Others noted that all aspects of the intervention were perceived to be enjoyable. For aspects of the program that participants least liked, a common response related to the frequency of the sessions, with participants’ noting the program was ‘too short’ and ‘wanting a session every week of the year, not every 2 weeks for a limited time only’. For aspects of the program that could be improved, participants again reported that they wanted weekly sessions, wider dissemination so more women could participate, a program for children, conduct the program again, and to also encourage participants to be more punctual.

### Health-related outcomes

Table 4 presents the results of the Wilcoxon signed-rank tests exploring the changes in physical activity levels, self-efficacy for physical activity, and anthropometric outcomes from baseline to post-intervention. There were no statistically significant changes in any health-related outcomes from baseline to post-intervention.

**Table 4:** Differences in health outcomes from the Wilcoxon signed-rank test

Outcome	Median (quartile 1–quartile 3)		Z	*p*
	Baseline	12 weeks		
Objectively-measured physical activity				
Moderate-vigorous physical activity 10 min bouts (min/week, n = 11)	38.33 (15.83–95.00)	13.5 (0.00–68.50)	− 1.68	0.11
Step count (n = 11)	5525.50 (4824.86–7154.00)	6243.57 (4383.40–7627.14)	− 0.27	0.83
Light physical activity (% wear-time, n = 11)	24.94 (18.28–29.85)	25.77 (19.73–29.78)	− 0.80	0.47
Moderate physical activity (% wear-time, n = 11)	4.78 (2.79–6.63)	4.59 (3.56–5.35)	− 0.89	0.41
Vigorous physical activity (% wear-time, n = 11)	0.09 (0.04–0.27)	0.09 (0.05–0.15)	− 0.27	0.83
Sedentary (% wear-time, n = 11)	72.07 (63.39–77.33)	67.14 (59.91–75.04)	− 1.42	0.18
IPAQ short-form				
Walking physical activity (MET-minutes/week, n = 14)	396.00 (288.75–643.50)	313.50 (264.00–915.75)	− 0.39	0.72
Moderate physical activity (MET-minutes/week, n = 11)	120.00 (40.00–720.00)	240.00 (0.00–480.00)	− 0.26	0.84
Vigorous physical activity (MET-minutes/week, n = 13)	0.00 (0.00–360.00)	240.00 (0.00–480.00)	− 1.19	0.27
Total physical activity (MET-minutes/week, n = 10)	933.50 (716.50–1653.00)	834.00 (384.50–1109.25)	− 1.07	0.32
Sitting time (hours/day, n = 9)	4.00 (2.75–8.50)	5.00 (2.00–7.25)	− 0.42	0.74
Self-efficacy for physical activity (0–90, n = 14)	49.00 (34.75–57.25)	51.50 (31.00–67.75)	− 0.04	0.99
Anthropometric outcomes				
Waist circumference (cm, n = 15)	93.55 (88.05–107.10)	99.10 (88.95–104.05)	− 1.53	0.13
Weight (kg, n = 15)	83.50 (70.75–91.03)	83.60 (71.20–91.18)	− 0.09	0.95
BMI (kg/m^2^, n = 15)	32.31 (28.25–36.14)	32.17 (28.90–36.93)	− 0.06	0.98

*MET* metabolic equivalents, *BMI*  body mass index

## Discussion

The purpose of this study was to test the feasibility and acceptability of a 12-week culturally tailored physical activity intervention for Arab-Australian women aged 35–64 years. The findings from this study contribute to the limited available evidence on health promotion initiatives specifically targeting Arab-Australians.

Recruiting those from CALD backgrounds to participate in research is a challenging process. Initial recruitment strategies for this study, which included face-to-face presentations by the first author, distribution of flyers, and social media posts did not generate much interest from potential participants. A potential explanation for the difficulties with recruitment in the initial stages of recruitment could be linked to a lack of trust from potential participants towards the study personnel, as some Arab migrant populations may be reluctant to engage with those not known to them [36, 37]. Using respected personnel or community members known to participants is an important strategy for recruitment and to help establish trust [36, 37, 54]. The second phase, which involved direct contact methods to potential participants who frequented the two community centres (intervention sites) by a community worker known to participants, was more efficient and successful in recruiting the desired number of participants for this study. Another strategy for recruiting Arab migrant populations is through word-of-mouth or snowballing approaches [36, 37, 54]. Similarly, many participants in the current study reported that they found out about the program through word-of-mouth. However, a limitation of this mode of recruitment is that it may be more likely to recruit women who frequent community centres or who are known to community workers. Given that some CALD populations may not be aware of available services [6], greater efforts targeting those who do not frequent community centres is required. A potential strategy to have a wider reach for recruitment of Arab populations could be to explore other trusted locations for recruitment such as local mosques or family physician settings.

The overall retention rate was 68%, and retention rates were higher at intervention site two (78%) in comparison to site one (62%). A potential explanation for the higher retention rate at site two could be due to it being a health centre, consequently the women who were recruited and participated at this centre may be more health conscious and inclined to complete the program in comparison to women who were recruited and participated at site one, which was a welfare organisation. Offering incentives to improve attendance rates may need to be considered when designing interventions for Arab-Australian women.

The intervention delivered at site one coincided, in part, with Ramadan. A previous study delivering a health promotion intervention for Arab-Americans scheduled an intervention during Ramadan without any reported issues [29]. However, a pilot physical activity intervention targeting South Asian Muslim women did not offer any sessions during Ramadan [55]. It has previously been reported that physical activity can be safely performed during Ramadan without posing any health risks [46]. As participants would be fasting during daylight hours, lighter-intensity exercise was scheduled during these sessions. A total of 11 of 15 (73%) participants attended the first of the two sessions that coincided with Ramadan, however, the subsequent session, which was 1 week before Eid had to be rescheduled due to limited participant availability. All but one participant at site one reported in the process evaluation that Ramadan was a barrier to attending intervention sessions. A potential explanation for Ramadan being perceived as a barrier could be due to a lack of time as a result of extra responsibilities during this period, such as preparation for evening meals (i.e., for the breaking of fast) and also preparation for Eid. Based on the feedback, it could be suggested that physical activity programs should avoid being scheduled during Ramadan. Alternatively, programs delivered in the lead up to Ramadan could have dedicated sessions, specifically focusing on Ramadan and health behaviours, such as physical activity, as previously done in an intervention targeting Arab-Americans [29]. However, it should not be discounted that many participants at site one were able to attend at least one session during Ramadan. As many Arab women may be unavailable after sunset due to the breaking of fast and acts of worship, and as physical activity should be performed year-round, physical activity programs for Arab-Australian women may be able to run during daylight hours in Ramadan, albeit with reduced face-to-face contact and lighter-intensity physical activity.

Comparing self-reported time in physical activity to objectively-measured physical activity suggests that participants overestimated their physical activity levels, which is a common issue associated with the short-form IPAQ [56]. As per the eligibility criteria, participants were ineligible if they reported engaging in 150 min or more of moderate-vigorous physical activity over five or more days, however self-reported baseline physical activity levels as indicated in the short-form IPAQ exceeded this amount. Overreporting associated with the short-form IPAQ is not uncommon, as findings from a systematic review indicated that self-reported physical activity levels from the short-form IPAQ were generally overestimated in comparison to objectively-measured physical activity [56]. Further, when assessing physical activity levels with the short-form IPAQ, participants were asked to take activities into consideration if they were performed in bouts of at least 10 min. A potential explanation for the overestimation of physical activity levels could be due to participants listing all physical activity they have performed irrespective of whether or not the physical activity was accrued with a minimum of 10-min bouts [57]. Another potential explanation could be that participants may report the same physical activity for more than one question [57]. In the future, a potential strategy to minimise the risk of overreporting would be to explain aspects of the questionnaire to participants beforehand (e.g., consider 10-min bouts only, each activity intensity is distinct) [57] or having an interviewer administer the questionnaire to participants to clarify inconsistencies in reporting.

As compliance with the use of accelerometers was low among participants in this study, ways of addressing this need to be built into future studies. Some participants did not have any data recorded on their assigned accelerometer and others did not wear the accelerometer for a sufficient number of days. A high level of compliance with accelerometers is important to obtain an accurate measure of the regular physical activity levels of participants [58]. Strategies to promote compliance could be to ask participants to complete activity logs for self-monitoring, phone call reminders, and also offering incentives for achieving minimum wear time [58].

To ensure the intervention was culturally acceptable for Arab-Australian women, formative research, with the use of participatory methods, was conducted to culturally tailor the intervention. Participatory methods are a key component of interventions targeting CALD populations [32]. The culturally tailored components included culturally-relevant content and intervention material, provision of gender-matched research assistants, delivery of the intervention at familiar sites, and gender-exclusive settings for physical activity. These aspects of the intervention were generally favoured by the participants as indicated in the process evaluation, which have been similarly reported in previous pilot physical activity interventions targeting Muslim women. For example, gender-exclusive settings were an important facilitator for participation and provided women with a sense of comfort [59, 60]. Additionally, engaging in programs with other members of the community was also reportedly a facilitator [59]. Although most participants agreed with the statement ‘Cofacilitation of the education sessions by a male facilitator did not bother me’, this statement received the lowest score and had the most variability in ratings among other culture-related intervention components. Although interested potential participants were aware that the program would be cofacilitated by a male and female Arab-Australian, we cannot rule out this being a potential deterrent for those who did not enrol in the study and those who did not complete the study. Future interventions targeting Arab-Australian women should consider employing only female facilitators, preferably of Arab ethnicity.

General program aspects that participants favoured included the group-based sessions, supportive facilitators, enjoyable and inclusive physical activity sessions, program structure, the venue, and the social aspect of the program. The findings from a previous pilot mosque-based physical activity intervention reported on the social benefits of such programs allowing women to interact socially and form relationships with new women [59]. Group-based settings are an important mode of delivering health education and help normalise conversations about health [61]. Additionally, having sessions within the local community can help facilitate attendance in physical activity programs [59]. Many participants, however, were not satisfied with only one session per fortnight as indicated in the process evaluation, with participants indicating that they would prefer weekly sessions. The rationale for the fortnightly scheduling was to allow time for participants to implement strategies learnt in each session, to promote accountability, and also due to time commitments as time is a commonly reported barrier for Arab-Australian populations [7, 22]. Participants were encouraged to connect with their friends to engage in physical activity in the weeks where there were no scheduled sessions, however, this was not monitored. Future interventions should look to incorporate stronger strategies to encourage participants to work together in-between scheduled sessions in order to facilitate progress. For example, this could include nominating a group champion or a highly respected group member who is responsible for organising and setting the types of activities to be performed for weeks in-between scheduled sessions. Additionally, interventions should consider offering class frequency options tailored to the needs of Arab-Australian women.

On average, there appeared to be favourable changes in outcomes showing indication of improvement among several outcomes from baseline to post-intervention. Additionally, data from the process evaluation suggested that all participants perceived their physical activity levels to have increased. However, there were no statistically significant differences in any outcomes as indicated by the Wilcoxon signed-rank tests. As this study was a feasibility and acceptability study with a small sample, it was not sufficiently powered to detect changes in outcomes.

As this study used a single-group pretest–posttest design and had a small sample, definitive conclusions regarding the efficacy of the intervention cannot be made from the findings. However, testing for preliminary efficacy was only exploratory in nature and a secondary purpose, with the primary focus on assessing feasibility and acceptability of the intervention. A limitation related to the generalisability of this study, was that the majority of the sample were Muslim women who frequented or engaged with community centres. Consequently, the findings of this study may not be generalisable to those Arab-Australians of other religions and women who do not regularly visit community centres. Another limitation is the use of the short-form IPAQ, which was initially chosen given its lower burden to participants compared to the long-form IPAQ. Due to overreporting physical activity and that this instrument does not distinguish household physical activity (a form of physical activity that is commonly reported among this population) from other forms of physical activity, instruments with a higher level of validity and that distinguish household physical activity from other forms are recommended for this population.

A strength of this study was the use of accelerometers for objective measures of physical activity levels. Another strength was that this study was culturally tailored to suit the needs and preferences of Arab-Australian women and included members of the target population throughout all stages of the intervention (i.e., conceptualisation to implementation). Adopting participatory methods in health interventions is crucial for intervention success and acceptability from the target population. Further, this study contributes to the limited available evidence towards health promotion among Arab-Australians, which highlights a number of important factors that need to be considered when developing and delivering health promotion interventions for Arab-Australian women. This study lays the foundations for future studies using more rigorous study designs.

## Conclusion

This study demonstrated the feasibility and acceptability of a 12-week culturally tailored physical activity intervention for Arab-Australian women aged 35–64 years. This study highlighted the important factors associated with feasibility (e.g., recruitment methods) and acceptability (e.g., session frequency) that need to be considered when developing physical activity interventions targeting Arab-Australian women. Further research is required using a larger sample and a randomised controlled trial design to examine the longer-term impact on physical activity, and to also examine ways of increasing intervention engagement and retention among Arab-Australian women.

## Data Availability

The datasets generated and/or analysed during the current study are not publicly available, but are available from the corresponding author on reasonable request.

## Abbreviations

BMI: Body mass index
CALD: Culturally and linguistically diverse
IPAQ: International physical activity questionnaire
MET: Metabolic equivalent
NSW: New South Wales
SCT: Social cognitive theory
SEE: Self-efficacy for exercise
SD: Standard deviation
US: United States
